# *15-Lipoxygenase-2* deficiency induces foam cell formation that can be restored by salidroside through the inhibition of arachidonic acid effects

**DOI:** 10.1515/biol-2025-1091

**Published:** 2025-04-29

**Authors:** Rong Huang, Xi Yong, Tingting Li, Huling Wen, Xing Zhou, Yichen Liao, Jun You, Chunlei Yu, Peng Xu, Yuquan Wang, Dan Wen, Tianqin Xia, Hao Yang, Yanqin Chen, Lei Xu, Xiaorong Zhong, Xianfu Li, Zhengmin Xu, Chunyang Zhou

**Affiliations:** Institute of Materia Medica, School of Pharmacy, North Sichuan Medical College, No. 234, Fujiang Road, Nanchong, Sichuan, 637000, China; Department of Vascular Surgery, Department of Nuclear Medicine, Radiotherapy Department, Department of Oncology, Department of Pharmacy, Affiliated Hospital of North Sichuan Medical College, Nanchong, Sichuan, China; Department of Pharmacy, Second Affiliated Hospital of North Sichuan Medical College, Nanchong, Sichuan, China; Translational Medicine Research Center, Institute of Hepatobiliary Research, School of Basic Medical Sciences, North Sichuan Medical College, Nanchong, Sichuan, China

**Keywords:** 15-lipoxygenase-2, foam cell formation, macrophages, arachidonic acid, salidroside

## Abstract

15-Lipoxygenase-2 (15-Lox-2) is one of the key enzymes in arachidonic acid (AA) metabolic pathway, which belongs to the unsaturated fatty acid metabolic pathway. This pathway is involved in the foam cell transformation of macrophages during the progression of atherosclerosis (AS). The role of salidroside (SAL) in cardiovascular diseases has been extensively studied, but its impact on macrophage foam cell formation has not yet been clearly clarified. We aimed to determine the effects of *15-Lox-2* deficiency on macrophage (Ana-1 cell) foam cell formation, and those of SAL on *15-Lox-2*-deficient macrophages. *15-Lox-2*-deficient macrophages were generated using short hairpin RNA. Results indicated that 15-Lox-2 expression in the aorta of atherosclerotic patients is lower than that of the normal group. Additionally, *15-Lox-2* deficiency dramatically promoted macrophage uptake of oxidized low-density lipoprotein (ox-LDL) and increased the Cyclin D1 level while dramatically decreasing caspase3 expression. Furthermore, inflammation, complement, and TNF-α signaling pathways, along with IL1α, IL1β, IL18, and Cx3cl1, were activated in *15-Lox-2*-deficient macrophages. These changes were alleviated by SAL through inhibiting AA effects, and the effects of AA on macrophages could be inhibited by SAL. Consistently, phospholipase A2-inhibitor arachidonyl trifluoromethyl ketone (AACOCF3) restored these changes. In summary, SAL reversed the effects of *15-Lox-2* deficiency on macrophages by inhibiting excessive AA and may be a promising therapeutic potential in treating atherosclerosis resulting from *15-Lox-2* deficiency.

## 1 Introduction

Atherosclerosis (AS), as one of the leading causes of cardiovascular disease, is an inflammatory and dyslipidemic disease [1,2]. Under normal physiological conditions, the body maintains a lipid metabolism homeostasis, many factors can disrupt this homeostasis, causing related diseases such as AS and hyperlipidemia [3]. Monocyte-derived macrophages excessively take up oxidized low-density lipoprotein (ox-LDL) to form foam cells and induce inflammation, which plays an essential role in atherogenesis [4,5]. Emerging studies carried out in cell or animal models of AS and patients with AS have demonstrated that excess polyunsaturated fatty acid could promote the onset and development of AS and increase the risk of cardiovascular disease [6]. Arachidonic acid (AA), a lipid mediator, is one of the most abundant and widely distributed polyunsaturated fatty acids in mammals [7]. AA produces various products through three enzymatic pathways, P-450 epoxygenase, cyclooxygenases, and lipoxygenases (LOXs). LOXs are involved in the biosynthesis of many lipid mediators [8]. Among the LOX family, 15-lipoxygenase-2 (*15-Lox-2*) shows the highest homology to murine *15-Lox-2* (also named Alox8) and lower identity to human *5-LOX* or *15-LOX-1* [9,10]. 15-LOX-2 specifically catalyzes the oxygenation of the 15th carbon (C15) of AA, producing 15(*S*)-hydroperoxy-eicosatetraenoic acid (15(*S*)-HpETE). 15(*S*)-HpETE can be reduced by glutathione peroxidase to form 15(*S*)-hydroxy-eicosatetraenoic acid (15(*S*)-HETE), which has more stable biological activity. [11,12]. 15-LOX-2 shows a tissue expression pattern that includes the lymph node, skin, lung, and prostate, and its disorders might contribute to dysfunction in these organs [13,14]. We have shown that *15-Lox-2* deficiency in preB cells might promote lymphomagenesis [15]. *15-Lox-2* acts as a suppressor gene in tumorigenesis [14,16]. Intriguingly, *15-Lox-2* is constitutively active in human monocyte-derived macrophages and participates in the atherosclerotic process [13,17,18]. Salidroside (SAL) is an active component extracted from plants of the genus *Rhodiola*, which is used in traditional Chinese medicine [19]. SAL has extensive pharmacological activities, such as antioxidant, anti-cancer, and anti-cardiovascular effects, mediated by repressing inflammation and oxidative stress [20–24]. However, the link between *15-Lox-2* and SAL in the progress of macrophage foam cell formation was poorly understood. Here, we aimed to investigate the mechanism of SAL on the improvement of *15-Lox-2* deficiency-induced macrophage foam cell formation.

## 2 Methods

### 2.1 Cell lines and tissue samples

The Ana-1 cell line and HEK 293T cells were purchased from Boster Biological Technology Co., Ltd. (China) and maintained in RPMI-1640 and Dulbecco’s modified eagle medium containing 10% fetal bovine serum, respectively. Cells were incubated in an incubator of 5% CO_2_ at 37°C. Seventeen patients (10 males and 7 females) with AS were recruited and underwent plaque resection at Affiliated Hospital of North Sichuan Medical College. Patients did not receive any preoperative medications and treatments.


**Informed consent:** Informed consent has been obtained from all individuals included in this study.
**Ethical approval:** The research related to human use has been complied with all the relevant national regulations and institutional policies and in accordance with the tenets of the Helsinki Declaration and has been approved by the Medical Ethics Committee of Affiliated Hospital of North Sichuan Medical College (IRB: 2024ER23-1).

### 2.2 Generating *15-Lox-2*-knockdown macrophages

To create 15-Lox-2 short hairpin RNA (shRNA), the appropriate 15-Lox-2 primers were cloned into the pMSCV-mir30-SV40-GFP retroviral construct. To avoid errors caused by the off-target effects of a single shRNA, we designed two independent shRNAs targeting 15-Lox-2 (sh15-Lox-2.1252 and sh15-Lox-2.2865) to observe the consistency of the phenotypes. Virus packaging and infection were performed as reported previously [25]. Cells stably expressing 15-Lox-2 shRNA were selected using G418.

### 2.3 Hematoxylin and eosin (HE) and immunohistochemistry staining

HE staining was performed as described previously [26]. Immunohistochemistry was used to assay 15-Lox-2 expression in tissues. After being boiled for 2 min to restore the antigen and blocked by 5% bovine serum albumin (BSA), the samples were incubated with the primary antibody (15-Lox-2) overnight at 4°C and further incubated with HRP-goat-anti-rabbit IgG for 1 h at 25°C.

### 2.4 Liquid chromatography–mass spectrometry (LC–MS)

A total of 5 × 10^6^ cells were extracted with chloroform/methanol (2:1, v/v) and washed with 0.9% saline. The lipid-containing chloroform phase was obtained and dried. We then added 100 μL of methanol containing the deuterium-labeled internal standard AA-d8 and 5-HETE-d8 (Cayman Chemical, USA). LC–MS analyses were conducted on the Agilent LC–MS system (USA). Chromatographic separation was achieved on an Agilent ZORBAX RRHD Eclipse XDB C18 column (2.1 mm × 100 mm, 1.8 µm particles) using a flow rate of 0.65 mL/min at 45°C during a 13 min gradient (0–12 min from 68% A to 20% A, 12–13 min 5% A) while using the solvents A, water containing 0.005% formic acid, and B, acetonitrile containing 0.005% formic acid. Electrospray ionization was performed in the negative ion mode using N^2^ at a pressure of 30 psi for the nebulizer with a flow of 10 L/min and a temperature of 300°C, respectively. Peak determination and peak area integration were performed with the MassHunter WorkStation software (Agilent, Version B.08.00).

### 2.5 Oil Red O and DiI staining

Cells were stained with Oil Red O (cat: # O8010, Solarbio, China) and the fluorescent probe DiI (cat: # C1036, Beyotime, China) according to the manufacturer’s protocol, respectively. Furthermore, the value of optical density (OD) was determined for DiI and 4′,6-diamidino-2-phenylindole (DAPI) using a microplate reader (Thermo Fisher Scientific, USA), respectively.

### 2.6 RNA-seq

Total RNA was sequenced using BGISEQ500, and the results were analyzed using 50-bp single-end reads. The reads were aligned to the reference genome (GRCm38) using STAR_2.6.0. Transcript abundance was normalized and measured in reads/fragments per kilobase per million mapped reads. DESeq2 was used to analyze differential gene expression. Genes with absolute fold-changes in expression levels greater than 1 and a false discovery rate of ≤0.05 were considered differentially expressed. The characteristic differences between samples were assessed using principal component analysis (PCA). Based on the designated clusters, gene set enrichment analysis (GSEA) was performed to statistically analyze similarities and differences between the two types of samples.

### 2.7 Proliferation assay

The cells were assayed for proliferation using a Cell-Light™ Edu Apollo643 *In Vitro* Kit (cat: # C10310-2, Ribobio, China) according to the manufacturer’s protocol and further detected using flow cytometry (Agilent, USA), and data were analyzed using FlowJo v10.

### 2.8 Apoptosis assay

Cell proliferation was assessed using an Annexin V PE Apoptosis Dtec Kit (cat: # 559763, BD Biosciences, USA) according to the instructions. Samples were analyzed using flow cytometry (BD, USA).

### 2.9 Western blot

Proteins were separated by 10% sodium dodecyl sulfate-polyacrylamide gel electrophoresis and then transferred to a polyvinylidene fluoride membrane. The membrane was blocked with 5% BSA and incubated with the appropriate primary antibodies (Table 1) overnight at 4°C, followed by incubation with the appropriate secondary antibodies for 1 h at room temperature. Immunoreactive proteins were detected using the Vilber Lourmat Imaging System (France).

**Table 1:** Antibodies used in WB

Antibodies	Source	Identification
15-Lox-2	Novus	Cat: # NBP2-92668
Caspase3	CST	Cat: # 9662S
CCND1	Abcam	Cat: # ab16663
β-Tubulin	Thermo Fisher Scientific	Cat: # MA5-16308

### 2.10 RT-qPCR

Total RNA was isolated from cells using RNAiso Plus reagent (cat: # 9109, Takara, China) and reverse-transcribed into cDNA using a RevertAid First Strand cDNA Synthesis Kit (cat: # K1622, Thermo Fisher Scientific). All primers (Table 2) were designed using https://pga.mgh.harvard.edu/primerbank/. RT-qPCR was performed using the LightCycler96 system (Roche, Switzerland).

**Table 2:** Primers used in RT-qPCR

Primers	Primer sequence
β-Actin	F: 5′-ATGGAGGGGAATACAGCCC-3′
β-Actin	R: 5′-TTCTTTGCAGCTCCTTCGTT-3′
Cx3cl1	F: 5′-ACGAAATGCGAAATCATGTGC-3′
Cx3cl1	R: 5′-CTGTGTCGTCTCCAGGACAA-3′
IL18	F: 5′-CTCTGTGGTTCCATGCTTTCT-3′
IL18	R: 5′-GTTTGAGGCGGCTTTCTTTG-3′
IL1α	F: 5′-CAGATCAGCACCTTACACCTAC-3′
IL1α	R: 5′-GAGATAGTGTTTGTCCACATCCT-3′
IL1β	F: 5′-GGCAGGCAGTATCACTCATT-3′
IL1β	R: 5′-GAAGGTGCTCATGTCCTCATC-3″

### 2.11 Statistical analysis

All experiments were performed three times independently. All data were analyzed by GraphPad Prism6.0. Data are shown as the mean ± standard deviation (SD). Comparisons between groups were analyzed using unpaired two-tailed *t*-tests and one-way analysis of variance. Differences were expressed as *p*-values; *p* < 0.05 was considered statistically significant. The number of samples or events in the study was denoted in the figure legends.

## 3 Results

### 3.1 The *15-Lox-2* expression decreased in AS

**Figure 1:**
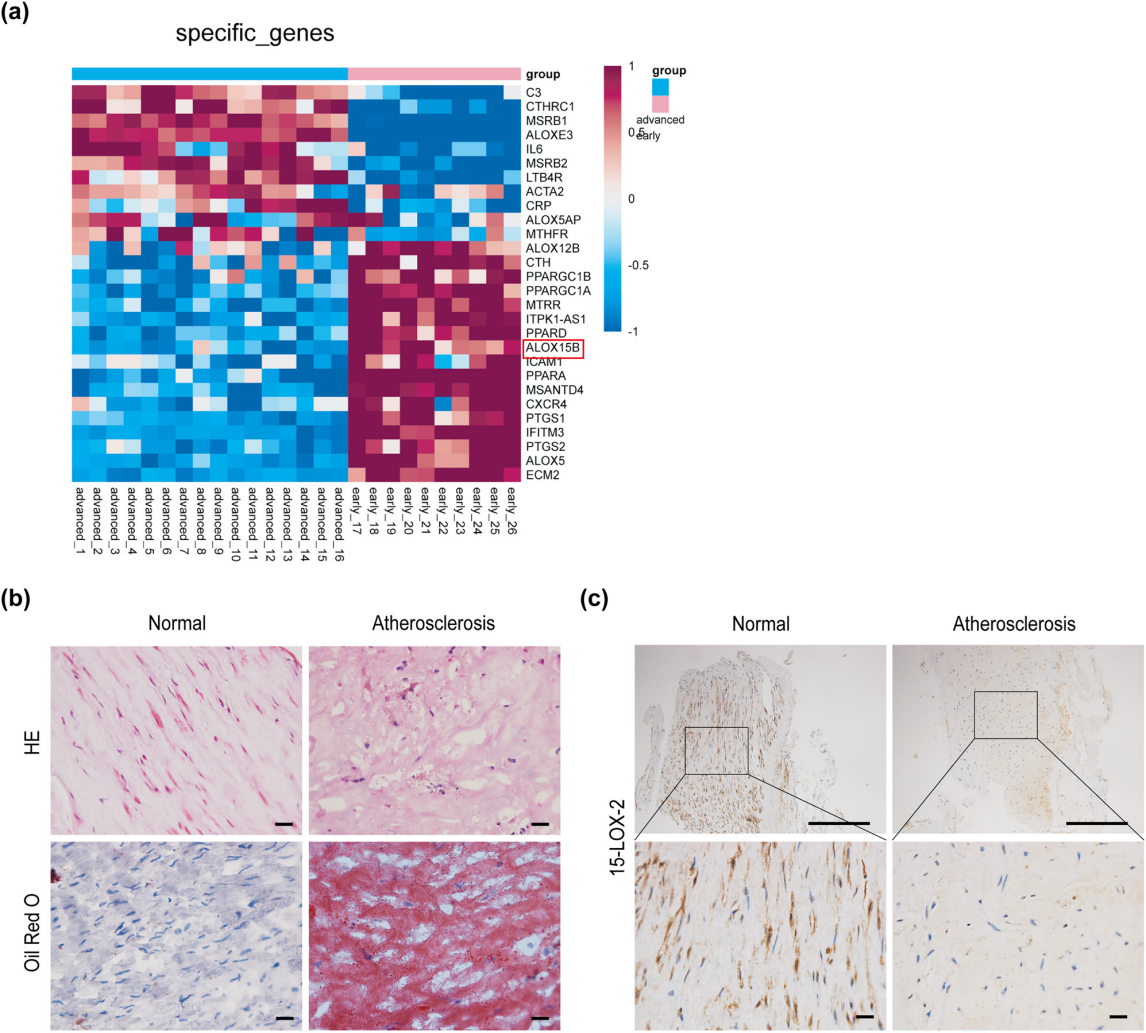
The expression of 15-Lox-2 decreased in advanced AS. (a) The differential expression of 15-Lox-2 in early and advanced AS. (b) The vessels of patients with AS were monitored by HE or Oil Red O staining, scale bar: 100 μm. (c) The 15-Lox-2 expression in vessels was detected by immunohistochemistry, scale bar: 100 μm.

Variance analysis was used to explore the expression of 15-Lox-2 in the different stages of AS. As shown in Figure 1a, the expression of 15-Lox-2 was significantly decreased in advanced AS as compared to normal, while increased in early AS. Subsequently, advanced AS endarterectomy specimens and non-atherosclerotic specimens were harvested (Figure 1b). Immunohistochemistry analyses showed that the 15-Lox-2 expression was significantly higher in atherosclerotic plaque than normal (Figure 1c).

### 3.2 *15-Lox-2* deficiency-induced macrophage foam cell formation

**Figure 2:**
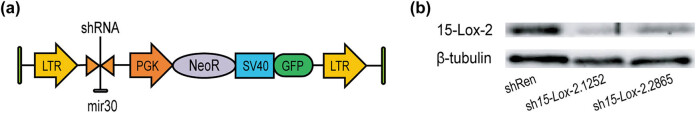
The model of sh*15-Lox-2* macrophages. (a) Schematic representation of virus vector; shRNAs were cloned into the backbone named mir30. (b) The knockdown efficiency of sh*15-Lox-2.1252* and sh*15-Lox-2.2865* was detected by WB, compared with shRen. shRen is used as a control due to its sequence that does not target any genes.

**Figure 3:**
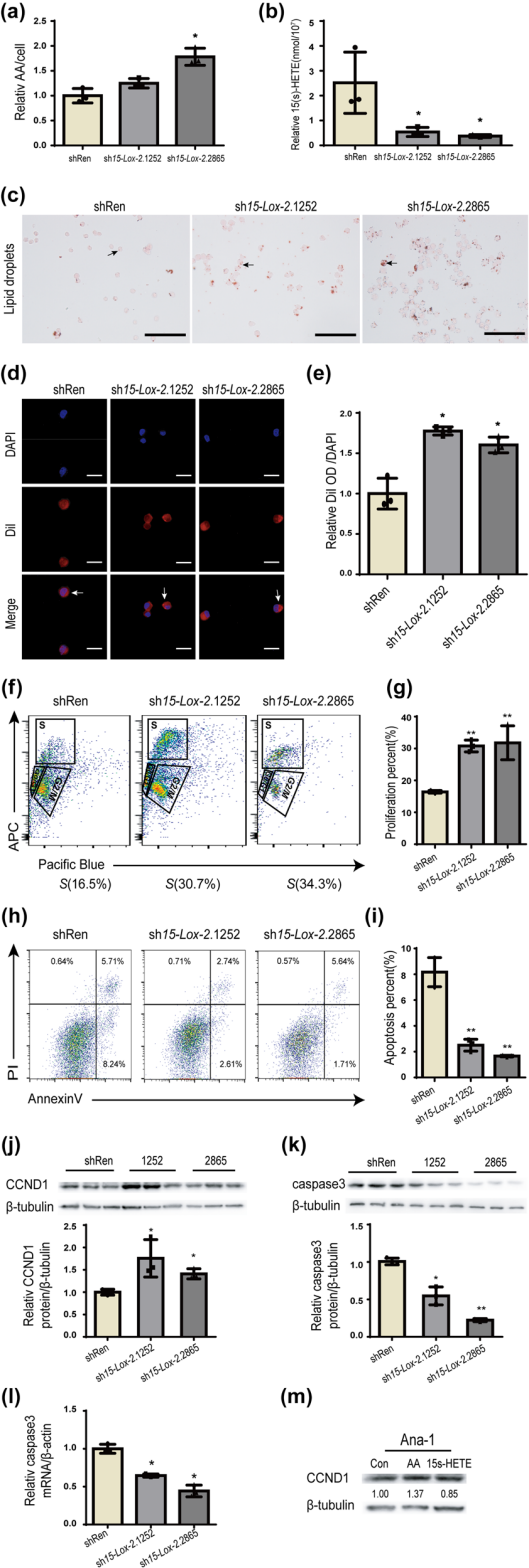
*15-Lox-2* deficiency promoted foam cell formation and survival of macrophages. (a) The levels of AA and (b) 15(*S*)-HETE were analyzed by mass spectrometry. (c) Representative images of Oil Red O staining images of cells, scale bar: 100 μm; black arrows show lipid droplets in cells. (d) Representative images of DiI staining images of cells, scale bar: 25 μm; white arrows show lipid droplets. (e) DiI and DAPI OD were determined using a fluorescence microplate reader. (f) and (g) The percentage of Edu-positive cells and (h) and (i) AnnexinV/7-AAD-positive cells was detected by flow cytometry and quantified by FlowJo V10. (j) The levels of CCND1 and (k) caspase3 were measured by WB and quantitated by ImageJ. (l) The levels of caspase3 mRNA were detected by RT-qPCR. (m) The CCND1 expression of Ana-1 cells treated with AA or 15(*S*)-HETE was detected by WB. Data are expressed as mean ± SD, *n* = 3, *p < 0.05, **p < 0.01 vs shRen.

To validate the role of *15-Lox-2* in macrophage foam cell formation, two independent 15-Lox-2 shRNAs (sh15-Lox-2.1252 and sh15-Lox-2.2865) and shRen were introduced into GFP and Neo vectors (Figure 2a). Then, the 15-Lox-2 shRNAs were introduced into Ana-1 macrophages to construct stable cell lines with *15-Lox-2* deficiency. As shown in Figure 2b, the expression of 15-Lox-2 in sh*15-Lox-2* macrophages was lower than that in shRen. Furthermore, the level of AA was higher in *15-Lox-2*-deficient macrophages than those in controls (Figure 3a), whereas 15(*S*)-HETE was decreased in *15-Lox-2*-deficient cells (Figure 3b).

As is known, macrophages can consume a substantial amount of ox-LDL and transform into foam cells, which is a key factor in atherosclerotic lesion progression [2]. To examine the phagocytosis of *15-Lox-2*-deficient macrophages, we monitored the uptake of ox-LDL in macrophages. As shown in Figure 3c and d, the cytoplasm of *15-Lox-2*-deficient macrophages was filled with ox-LDL, whereas few ox-LDL were observed in controls. The fluorescence OD results also showed that ox-LDL was markedly more abundant in *15-Lox-2*-deficient macrophages than in controls (Figure 3e). These data suggested that the phagocytosis of ox-LDL by *15-Lox-2*-deficient macrophages was enhanced, and macrophages tended to form foam cells following *15-Lox-2* deficiency.

We found that the 15-Lox-2-deficient macrophages have a faster proliferation than those of controls within the entire culture duration. As a result, we further assessed the effect of *15-Lox-2* deficiency on Ana-1 proliferation and apoptosis, respectively. The percentages of cells in the S phase were increased in *5-Lox-2-*deficient macrophages when compared to controls (Figure 3f and g). Additionally, the percentages of early apoptotic macrophages were lower in *15-Lox-2*-deficient macrophages than in controls (Figure 3h and i). Next, we tested the levels of proteins related to cell survival. The levels of Cyclin D1 (CCND1) were measured because an increase in CCND1 would indicate cell commitment to proliferation through cellular G1/S transition. *15-Lox-2*-deficient macrophages showed a significant increase in CCND1 expression (Figure 3j), indicating that the deficiency of *15-Lox-2* promoted cellular proliferation. Caspase3 encodes a cysteine protease that has been linked to the promotion of cell apoptosis. As shown in Figure 3k, compared with those in controls, caspase3 levels were significantly reduced in *15-Lox-2*-deficient macrophages. Caspase3 mRNA level also decreased in sh*15-Lox-2* macrophages relative to that in controls (Figure 3l). These data suggested that *15-Lox-2* deficiency might promote macrophage activity. Moreover, the expression of CCND1 in Ana-1 cells treated with AA was upregulated, but it was downregulated in cells treated with 15(*S*)-HETE (Figure 3m), which suggested that the product and substrate of 15-Lox-2 may impact the fate of macrophages.

### 3.3 *15-Lox-2* deficiency is associated with enhanced inflammation-related pathways

**Figure 4:**
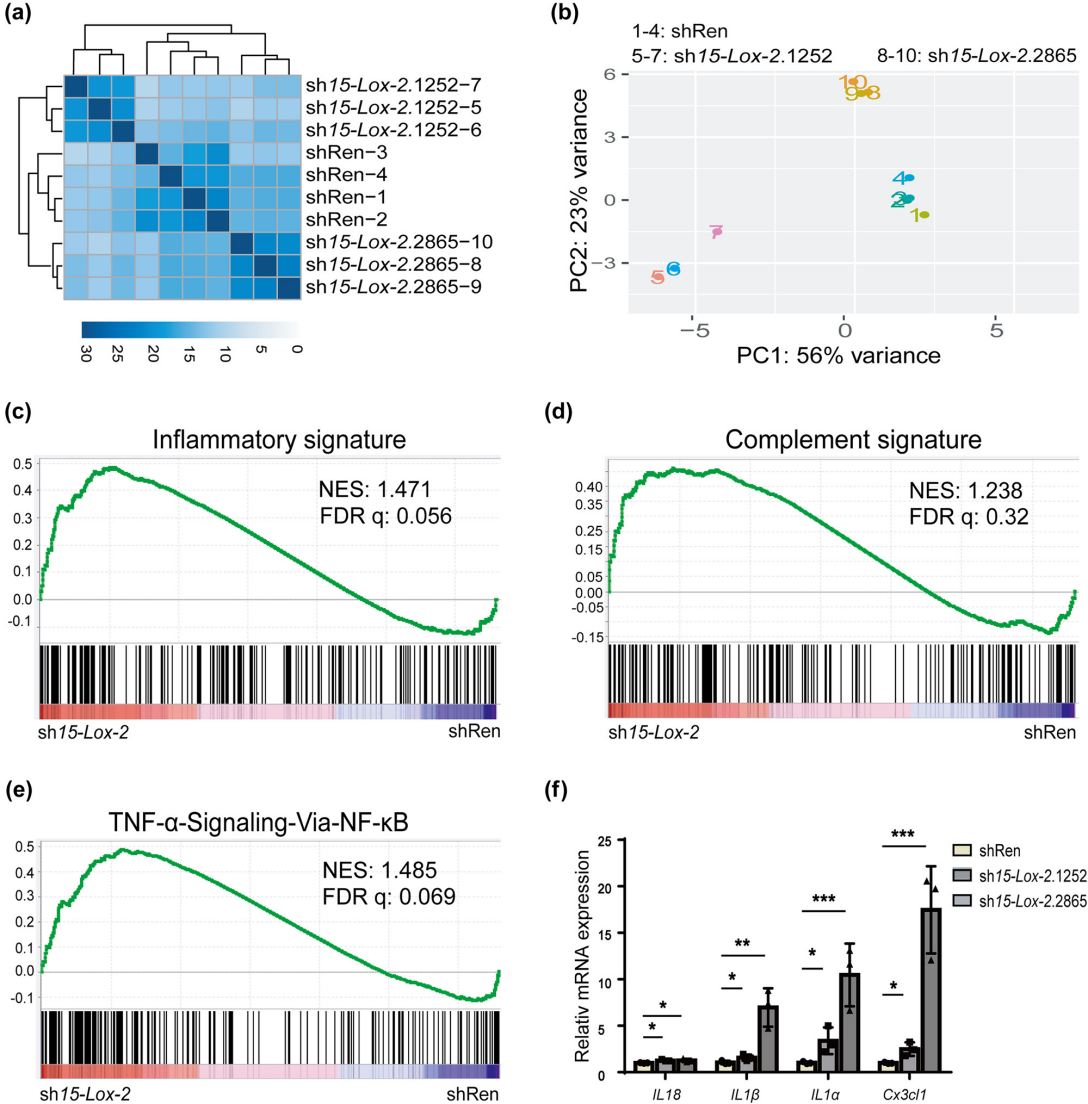
*15-Lox-2* deficiency associated with activation of inflammation-related signaling pathways. (a) Unsupervised clustering of *15-Lox-2*-deficient macrophages. (b) PCA of *15-Lox-2*-deficient macrophages. (c) GSEA of the hallmark gene sets showed positive enrichment of inflammatory response, (d) the complement signaling pathway, and (e) the TNF-α signaling via NF-κB. NES, normalized enrichment score; FDR, false discovery rate. (f) The mRNA level of IL18, IL1α, IL1β, and Cx3cl1 was performed by RT- qPCR. Results are presented as the mean ± SD, *n* = 3, *p < 0.05, **p < 0.01, ***p < 0.001 vs shRen.

To further explore the mechanisms of *15-Lox-2* in macrophage function, RNA-seq was performed to analyze the transcriptomes of Ana-1 macrophages expressing sh*15-Lox-2* and shRen. Both unsupervised clustering and PCA plots showed that macrophages expressing sh*15-Lox-2*.1252 and sh*15-Lox-2*.2865 were grouped together and clearly separated from shRen cells, indicating that the off-target effects of these two shRNAs were minimal (Figure 4a and b). Notably, compared with those in the controls, multiple gene sets related to inflammation, the complement pathway, and the TNF-α signaling pathway were activated in sh*15-Lox-2*-expressing cells (Figure 4c–e). RT-qPCR results revealed that IL18, IL1α, IL1β, and Cx3cl1, all related to the pathways identified, were upregulated in *15-Lox-2*-deficient macrophages (Figure 4f). These data indicated that *15-Lox-2* deficiency regulates the inflammatory response in Ana-1 macrophages.

### 3.4 *15-Lox-2* deficiency leads to an increase in AA, which may result in macrophage dysfunction

**Figure 5:**
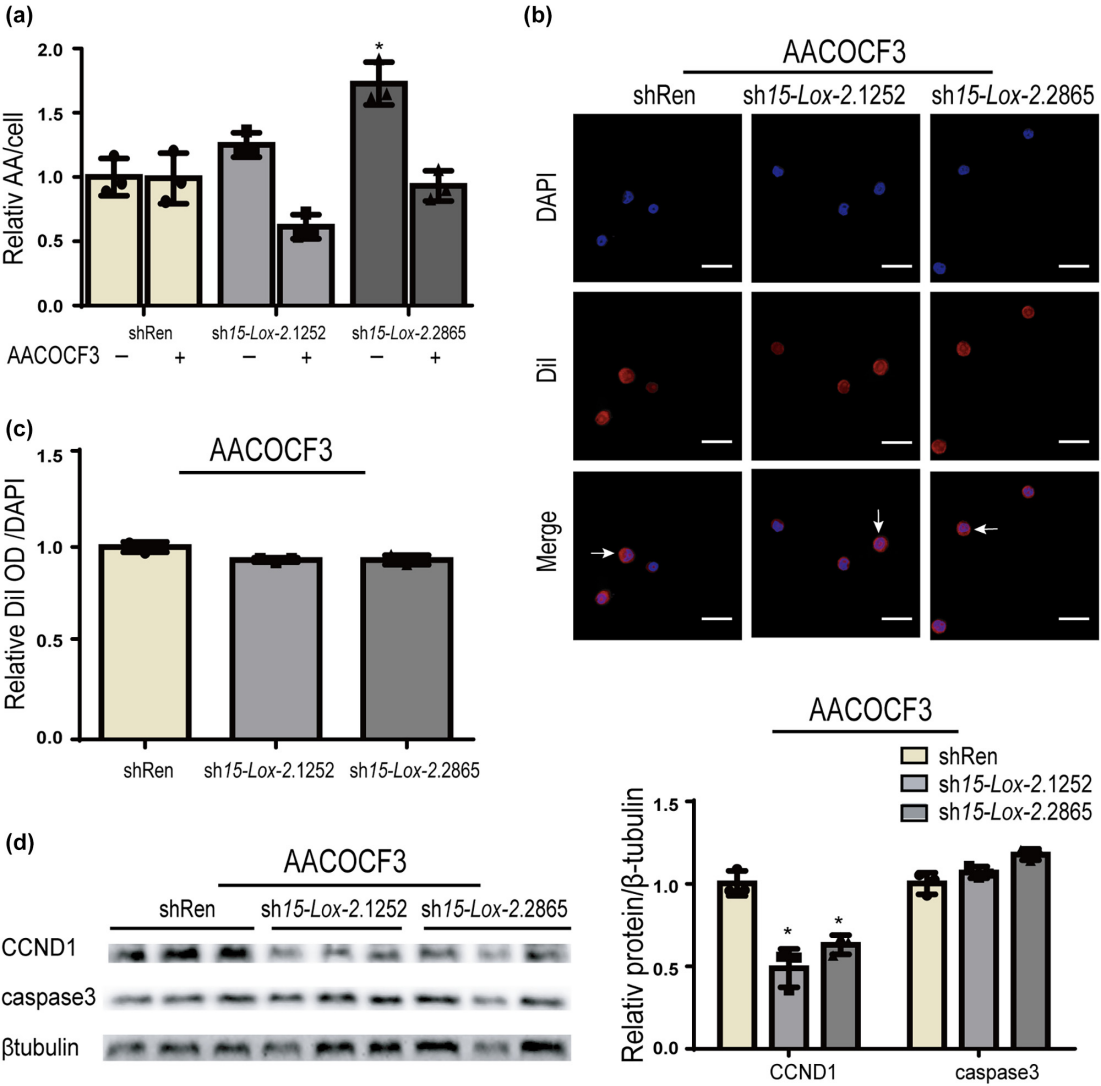
AACOCF3 alleviated the abnormal function of *15-Lox-2*-deficient macrophages. (a) The levels of AA were analyzed by mass spectrometry. (b) Representative DiI staining images of cells, scale bar: 25 μm. White arrows show lipid droplets located in the cytoplasm. (c) DiI and DAPI OD were determined using a fluorescence microplate reader. (d) The CCND1 and caspase3 expression of cells treated with AACOCF3 were detected by WB and quantitated by ImageJ. Results are presented as the mean ± SD, *n* = 3, *p < 0.05, vs shRen.

AA, a common 20-carbon polyunsaturated fatty acid, is mainly located in the plasma membrane and plays a remarkable role in the progress of AS [27]. Based on the above research shown in Figure 3a, b, and j, we presume that the effect of *15-Lox-2* deficiency on macrophages may be attributed to its metabolic substrate called AA. Then, arachidonyl trifluoromethyl ketone (AACOCF3) was used to treat macrophages. As shown in Figure 5a, after treatment with AACOCF3, AA level in *15-Lox-2*-deficient macrophages was decreased near to the level in controls. Furthermore, AACOCF3 decreased the fluorescence intensity of DiI-ox-LDL in *15-Lox-2*-deficient macrophages to a level close to that of the controls (Figure 5b and c), suggesting that AACOCF3 could ameliorate the phagocytosis of *15-Lox-2*-deficient macrophages via decreasing AA level. Additionally, the increased CCND1 expression of *15-Lox-2*-deficient macrophages was attenuated by AACOCF3, and the reduced caspase3 expression was increased by AACOCF3 to near controls (Figure 5d). These results suggested that the cell activity and phagocytosis were activated in an AA-dependent manner in Ana-1 macrophages.

### 3.5 SAL alleviated the phagocytosis of *15-Lox-2*-deficient macrophages by inhibiting AA effects

**Figure 6:**
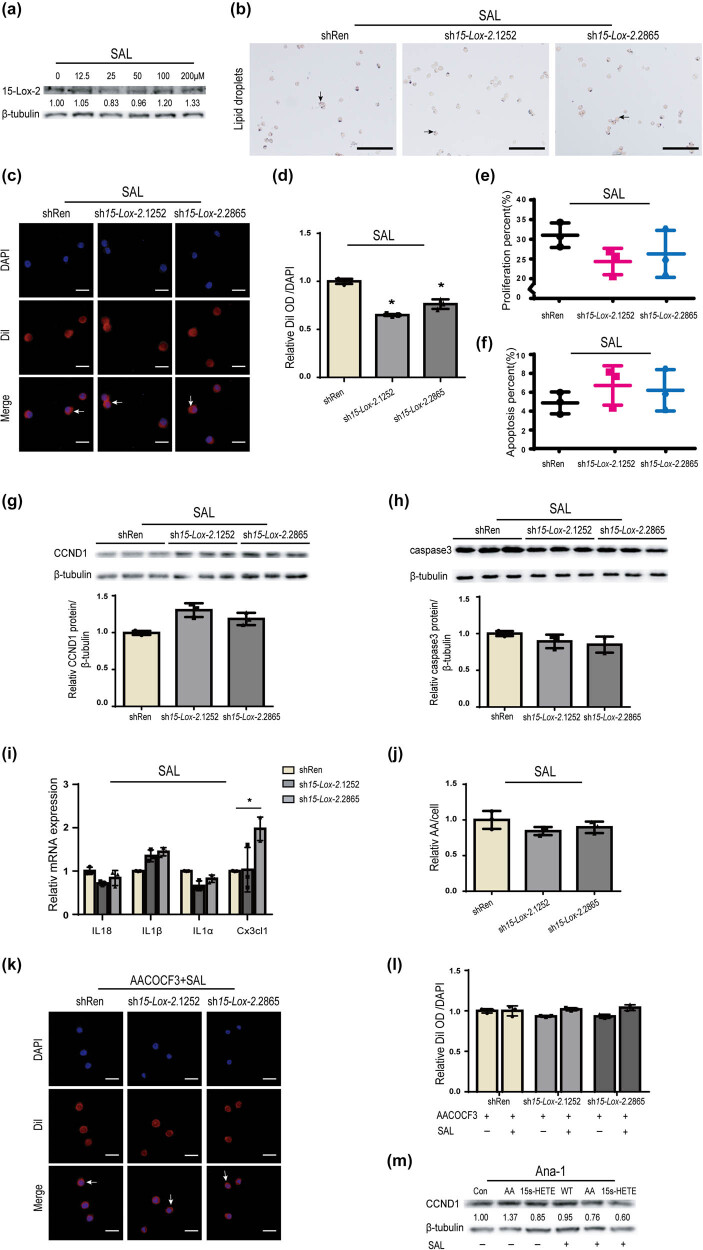
SAL reversed the phagocytosis of macrophages activated by *15-Lox-2* deficiency via acting on AA. (a–j) Macrophage-derived foam cells were treated with SAL. (a) The 15-Lox-2 expression of Ana-1 treated with 0–200 μM SAL was measured by WB. (b) Representative Oil Red O staining images of cells, scale bar: 100 μm; black arrows show lipid droplets in cells. (c) Representative DiI staining images of cells, scale bar: 25 μm. White arrows show lipid droplets located in the cytoplasm. (d) DiI and DAPI OD were determined using a fluorescence microplate reader. (e) The percentage of Edu-positive cells and (f) AnnexinV/7-AAD-positive cells was detected by flow cytometry and quantified by FlowJo V10. (g) The CCND1 and (h) caspase3 expression of cells were determined by WB and quantitated by ImageJ. (i) The levels of IL18, IL1α, IL1β, and Cx3cl1 mRNA were detected by RT-qPCR. (j) The levels of AA were tested by Mass Spectrometry. (k) Representative DiI staining images of cells co-treated with AACOCF3 and SAL, scale bar: 25 μm. White arrows show lipid droplets located in the cytoplasm. (l) DiI and DAPI OD were determined using a fluorescence microplate reader. (m) The levels of CCND1 in macrophages treated with AA, 15(*S*)-HETE, SAL, SAL plus AA, or SAL plus 15(*S*)-HETE were performed by WB. Results are presented as the mean ± SD, *n* = 3, *p < 0.05 vs shRen.

SAL has been shown to exert various pharmacological effects, including antioxidative stress and anti-inflammatory properties [28–30]. SAL can reduce *de novo* lipogenesis to attenuate AS [29]. The dosage of SAL selected in this study was based on its dose-dependent effect of SAL on the expression of 15-Lox-2 in Ana-1 cells and the results of the cell viability assay (Figure 6a) [29]. As shown in Figure 6b and c, the levels of ox-LDL observed in the *15-Lox-2*-deficient macrophages were close to those in shRen macrophages after treatment with SAL. The fluorescence OD also showed that ox-LDL in *15-Lox-2*-deficient macrophages were decreased to similar levels observed in controls (Figure 6d). These results suggested that SAL could alleviate the effect of *15-Lox-2* deficiency on phagocytosis of macrophages. Furthermore, after treatment with SAL, the increased proliferation of *15-Lox-2*-deficient macrophages was attenuated by SAL (Figure 6e). The reduced apoptosis of *15-Lox-2*-deficient macrophages was dramatically restored to normal levels by SAL (Figure 6f). Similar results were obtained from the analysis of proteins associated with proliferation and apoptosis in *15-Lox-2*-deficient macrophages treated with SAL. The upregulation of CCND1 expression and the decline in caspase3 protein caused by *15-Lox-2* deficiency were significantly reversed, returning to levels close to those in controls (Figure 6g and h). These results revealed that SAL may restore the abnormal cellular activity of *15-Lox-2*-deficient macrophages. Moreover, the mRNA levels of IL18, IL1α, IL1β, and Cx3cl1 in macrophages treating with SAL, which were all restored levels similar to those in controls (Figure 6i), indicating that SAL significantly decreased inflammatory response induced by *15-Lox-2* deficiency.

To determine whether SAL could decrease AA levels that were increased in *15-Lox-2*-deficient macrophages, AA levels were detected. After treatment with SAL, the AA levels in the *15-Lox-2*-deficient macrophages were decreased and got close to those in controls (Figure 6j). Moreover, after treating with both SAL and AACOCF3, there were no differences in ox-LDL density between controls and *15-Lox-2*-deficient macrophages, which were similar to that of cells treated with AACOCF3 alone (Figure 6k and l), indicating that SAL may inhibit the activity of phospholipase A2 (PLA2), thereby exerting its pharmacological effects. Subsequently, the CCND1 expression of macrophages treated with SAL alone or in combination with AA or 15(*S*)-HETE was tested by WB. As shown in Figure 6m, AA alone could upregulate the expression of CCND1 in cells. In contrast to AA, SAL combined with AA or 15(*S*)-HETE downregulated the expression of CCND1 in macrophages, indicating that SAL not only decreased the expression of CCND1 increased by AA but also enhanced the effects of 15(*S*)-HETE on CCND1 expression. All the data indicated that SAL may ameliorate the changes in macrophages caused by *15-Lox-2* deficiency via inhibiting AA effects.

## 4 Discussion

In this study, we found that *15-Lox-2* deficiency was prone to undergo foam cell formation and inflammatory response. SAL restored the changes in macrophages caused by *15-Lox-2* deficiency via inhibiting AA effects.

Macrophages are now known to have diverse and context-dependent functions in a variety of pathophysiological settings [31]. There is a rapidly growing interest in understanding how metabolic process-related genes, including lipoxygenases, can affect the appropriate activation of macrophages to enable host defense mechanisms. Multiple studies proved that 15-LOX-2 participated in various functions of macrophages, such as playing a key role in cancer and diseases of lipid metabolism [17,32]. Moreover, lipids regulate the inflammatory responses and phagocytosis of macrophages [33,34]. However, little is known about the importance of 15-LOX-2 and its relationship to physiological events in macrophage foam cell formation. In this study, using the loss-of-function way and transcriptomics approach, we highlighted the fact that the *15-Lox-2* deficiency had an impact on the phagocytosis of ox-LDL in macrophages, which might impact foam cell formation during the development of AS.

15-LOX-2 was found to affect the development of tumors through the regulation of AA levels in cells to impact tumor cell apoptosis and proliferation [35]. Here, we found that *15-Lox-2* deficiency increased AA levels in cells, inhibited apoptosis, and promoted the proliferation of macrophages. It has also been reported that 15-Lox-2 products (15(*S*)-HETE) might suppress immunosuppressive properties of ovarian tumor-associated macrophages and markedly inhibit the growth of tumor cells [36]. We found that 15(*S*)-HETE might downregulate the expression of the proliferative protein CCND1, but AA had the opposite effects on those proteins. It is known that AA is mainly located in the cell membrane and released by PLA2 [37,38]. Here, the AA level of *15-Lox-2*-deficient macrophages was decreased close to those of controls by the PLA2 inhibitor AACOCF3. The phagocytosis induced by ox-LDL in *15-Lox-2*-deficient macrophages was moderated by AACOCF3, suggesting that AA acted as a promoter in phagocytosis of 15-Lox-2-deficient macrophages, which was consistent with previous research on the role of AA in promoting atherosclerotic onset and progression [39]. Environmental and Intrinsic stimulation activates LOXs to produce significant amounts of downstream eicosanoids, such as leukotrienes (LTs) and lipoxins (LXs), and many aspects of the inflammatory response are regulated by LTs and LXs [40,41]. We showed that *15-Lox-2* was a crucial anti-inflammatory regulator, the down-regulation of *15-Lox-2* expression served as a positive feedback mechanism to activate inflammatory, complement, and TNF-α signaling pathways. All these results indicated that *15-Lox-2* deficiency might increase levels of AA to activate inflammatory response in macrophages.

Statins are the primary medicines used in clinical settings for the treatment of AS [42]. The anti-atherosclerotic effect of Statins is achieved by reducing cholesterol through the competitive inhibition of HMG-CoA reductase, the rate-limiting enzyme of endogenous cholesterol synthesis [43,44]. Therefore, statins are commonly prescribed as lipid-reducing medications [45]. However, statin therapy has limited effectiveness on various AS conditions due to adverse reactions and application constraints [46]. SAL, a safe medication, possesses antioxidant and anti-inflammatory properties and has been used for a long time to prevent aging and cardiovascular diseases [29,30]. This study showed that SAL restored abnormal phagocytosis and survival among inflammatory pathways and related genes (IL18, IL1α, IL1β, and Cx3cl1) of macrophages caused by *15-Lox-2* deficiency. Moreover, our previous study demonstrated that SAL had low toxic effects and significant pharmacological effects on macrophages, as well as exhibited anti-atherosclerotic effects *in vivo* [29], which was consistent with literature supporting the protective effect of SAL on AS [24,47]. Otherwise, previous studies have shown that omega-3 fatty acids possess immunomodulatory, anti-inflammatory, anti-platelet, and vascular protective effects in patients with AS [48,49]. However, there are many debates regarding the role of omega-3 fatty acids in cardiovascular disease [50,51]. Therefore, SAL may be a novel strategy to treat patients with AS who cannot effectively respond to other known anti-atherosclerotic medicines. Here, we found that SAL not only reversed the effects induced by AA but also enhanced the effects of 15(*S*)-HETE in macrophages. Notably, there were minimal differences in phagocytosis between *15-Lox-2*-deficient macrophages treated with both SAL and AACOCF3 and cells treated with AACOCF3 alone. These data indicated that SAL reversed the dysfunction of macrophages caused by *15-Lox-2* deficiency by inhibiting the effects of AA and blocking PLA2 activity. Some natural medicinal ingredients also exerted anti-atherosclerotic effects by inhibiting the secretion of AA and the inflammatory response[52], suggesting that the AA metabolic pathway may be an important avenue for natural medicine in AS treatment. AS, a chronic disease, primarily involves inflammatory response and disorders of lipid metabolism. Long-term use of medications is associated with numerous adverse reactions and limited efficacy [53]. Due to its extensive and effective pharmacological effects, low cost, and minimal side effects, SAL is anticipated to be utilized clinically as a foundational drug for the treatment of AS.

This study highlighted that *15-Lox-2* deficiency promoted macrophage foam cell formation, which could be alleviated by SAL via inhibiting the AA effects. SAL might be a promising therapeutic strategy to treat AS resulting from *15-Lox-2* deficiency. Nonetheless, our experiments were mainly carried out *in vitro*, accurate and comprehensive experiments need to be performed to further verify our results.
